# Monopolar Detection Thresholds Predict Spatial Selectivity of Neural Excitation in Cochlear Implants: Implications for Speech Recognition

**DOI:** 10.1371/journal.pone.0165476

**Published:** 2016-10-31

**Authors:** Ning Zhou

**Affiliations:** Department of Communication Sciences and Disorders, East Carolina University, Greenville, NC, United States of America; University of California Irvine, UNITED STATES

## Abstract

The objectives of the study were to (1) investigate the potential of using monopolar psychophysical detection thresholds for estimating spatial selectivity of neural excitation with cochlear implants and to (2) examine the effect of site removal on speech recognition based on the threshold measure. Detection thresholds were measured in Cochlear Nucleus^®^ device users using monopolar stimulation for pulse trains that were of (a) low rate and long duration, (b) high rate and short duration, and (c) high rate and long duration. Spatial selectivity of neural excitation was estimated by a forward-masking paradigm, where the probe threshold elevation in the presence of a forward masker was measured as a function of masker-probe separation. The strength of the correlation between the monopolar thresholds and the slopes of the masking patterns systematically reduced as neural response of the threshold stimulus involved interpulse interactions (refractoriness and sub-threshold adaptation), and spike-rate adaptation. Detection threshold for the low-rate stimulus most strongly correlated with the spread of forward masking patterns and the correlation reduced for long and high rate pulse trains. The low-rate thresholds were then measured for all electrodes across the array for each subject. Subsequently, speech recognition was tested with experimental maps that deactivated five stimulation sites with the highest thresholds and five randomly chosen ones. Performance with deactivating the high-threshold sites was better than performance with the subjects’ clinical map used every day with all electrodes active, in both quiet and background noise. Performance with random deactivation was on average poorer than that with the clinical map but the difference was not significant. These results suggested that the monopolar low-rate thresholds are related to the spatial neural excitation patterns in cochlear implant users and can be used to select sites for more optimal speech recognition performance.

## Introduction

One of the major factors that limit the processing of the electrical pulsatile stimulation with modern multichannel cochlear implants (CI) is channel interaction. Each electrode is assigned with a specific band of frequency information and should ideally stimulate an independent group of auditory fibers, maintaining the tonotopic organization of frequency coding. In reality, the electrodes often excite broader areas in the cochlea than desired, resulting in spatial overlapping in the neural excitation between channels. This explains why speech recognition saturates in quiet when the number of activated electrodes increases beyond eight [1], and why CI users do not demonstrate better performance in fluctuating noises than in steady state noise as normal-hearing listeners do [2]. Channel interactions are attributed to several factors related to pathology or position of the inserted electrode array. Electrodes located in cochlear regions with significant loss of spiral ganglion cells must stimulate a broad tonotopic region to recruit sufficient excitable neurons. Regions with high tissue impedance or abnormal anatomy might similarly require high current for stimulation resulting in spread of excitation. Broad current spread might also be produced by electrodes that are located far from the target nerve fibers. Because the variation patterns in pathology and electrode position are considerably different across the implanted ears [3–4], the neural spatial excitation pattern is also expected to be ear specific and site specific within ears.

It is important to develop clinically applicable methods that allow a time efficient estimation of the spatial neural excitation pattern along the array, because speech recognition might be improved if stimulation strategy can be customized to avoid regions that produce excessive spread of neural excitation. The goal of the current study was to investigate the potential of using a rather simplistic psychophysical method to measure spatial selectivity of neural excitation: the detection thresholds. In CI stimulation, the electrodes can be configured in several ways. In monopolar stimulation, the current is returned to one or two remote ground electrodes. In other stimulation modes (e.g., bipolar, tripolar, or partial tripolar), all or partial current is returned to one or more of the intracochlear electrodes on the electrode array, creating a more narrow current field compared to monopolar stimulation. In clinical mapping, detection thresholds are commonly measured in monopolar stimulation, where current is returned to ground electrodes outside the cochlea. It is also the only electrode configuration that is available in all implant device types.

We hypothesized that monopolar threshold can be used to estimate spatial selectivity of neural excitation, provided that neural response to the stimulus is minimally constrained by temporal inhibitive factors. In pulsatile stimulation, excitability of the single auditory fibers reduces as the rate or duration of the stimulus increases. When the stimulus is of relatively high-rate (1000 pps > rate >100 pps), after responding to a supra-threshold pulse in the pulse train, the probability of a neuron generating another action potential to the successive pulse depends on the neuron’s absolute and relative refractory period. The absolute refractory period measured in single neurons of cats is approximately 0.33 ms [5]. The relative refractory period can vary considerably across neurons and extends to a few milliseconds [5–6]. When the neurons are in a refractory status, they are said to have adapted to its prior activity. The neurons, however, can also adapt even though there is a lack of activity. If a pulse in the pulse train was below neural threshold and did not elicit an action potential, and if the cell membrane potential had returned back to rest by the time the following pulse occurred, the sub-threshold pulse would suppress the neuron’s excitability to the second pulse even though its amplitude was above neural threshold [7]. The sub-threshold pulse is said to desensitize the neuron, a mechanism known as sub-threshold adaptation that has been observed in human subjects with CIs [8]. It is important to distinguish this effect from the facilitative effect of accumulative partial depolarization, where the sub-threshold pulse partially depolarizes neural membrane and facilitates its response to the subsequent pulse. The charge on the cell membrane would only accumulate and facilitate detection if the pulses were less than 400 μs apart [6, 9–10]. Another factor that might reduce neural excitability is spike-rate adaptation, where neural activity reduces over the course of stimulus duration [11–12]. Spike-rate adaptation is designed to reduce redundancy in neural representation of repetitive stimulation. Because the auditory nerve fibers can vary considerably in their recovery constant of refractoriness and the various forms of adaptation especially with pathology (e.g., [7, 13]), it can be a source that produces variation in thresholds for high-rate and/or long-duration stimuli that is not necessarily related to the spatial patterns of neural excitation. More importantly, consider a scenario where two stimulation sites contrast greatly in the extent of current spread. Threshold at the stimulation site with greater current spread, due to for example distant stimulation, would be higher than that with a narrower stimulation pattern [4, 14]. We predict however that this threshold difference would diminish or disappear if stimulus used for measuring the threshold is increased in stimulation rate. The reasoning is that detection threshold reduces with a significantly steeper slope with increasing stimulus rate in a broader stimulation pattern [15]. As stimulation rate increases, neurons at the more periphery locations of a broad current field might be activated when the excitability of the neurons in the center of the current field is reduced, whereas in a narrow current field, the number of neurons that are available to be further recruited as rate increases might be relatively small [15]. Therefore, a broad stimulation pattern might in fact facilitate detection. Given these considerations, it was hypothesized that the differences in the neural spatial excitation patterns would be best captured by threshold for detecting pulse trains of low-rates and that the thresholds would be systematically less predictive of spatial selectivity as detection involves more temporal factors.

The second aim of the study was to use monopolar detection thresholds to estimate the spatial neural excitation patterns along the electrode array and examine if speech recognition would improve if the stimulation sites estimated with broad stimulation patterns were deactivated. As discussed earlier, channel interaction is thought to be one of the greatest obstacles for speech understanding with CIs, particularly in noise. We hypothesized that deactivating stimulation sites with broad spread of neural excitation would improve speech recognition, based on the assumption that speech information is coded better with a small number of independent channels than with a large number of channels that interact.

The results of the study indicate that both hypotheses were true that when neural responses to the stimulus were not limited by the temporal inhibitive factors, monopolar detection thresholds predicted the spatial pattern of neural excitation and that site selection based on the threshold measure significantly improved speech recognition.

## Materials and Methods

### Subjects and hardware

Ten postlingually deafened adult subjects with the Cochlear Nucleus^®^ devices participated in the study. Some of the subjects were sequentially bilaterally implanted (see subject demographic information in Table 1). Eleven ears were tested in Experiment 1, where the relationship between spatial selectivity and monopolar thresholds were examined. S8R had zero speech recognition in noise and in quiet and therefore did not continue with the speech recognition testing in Experiment 2. Two additional ears were recruited for Experiment 2, resulting a total *N* of 12. All subjects provided written informed consent before participating in the study. This study was approved by the East Carolina University institutional review board.

**Table 1:** Subject demographic.

Subject number	Gender	Age (yrs)	Duration of implant use (yrs)	Duration of deafness (yrs)	Device type	Participated experiment
S1L	M	75.3	12.2	0.1	CI24R(CS)	Exp 1 & 2
S1R	M	75.3	6.2	6.0	CI24RE (CA)	Exp 1 & 2
S2L	F	48.4	2.0	41.5	CI24RE (CA)	Exp 1 & 2
S2R	F	48.4	1.9	41.5	CI24RE (CA)	Exp 1 & 2
S4L	F	54.8	2.4	0.4	CI24RE (CS)	Exp 2
S5L	F	79.8	4.3	3.5	CI512	Exp 1 & 2
S6R	F	82.3	2.1	65.2	CI24RE (CS)	Exp 1 & 2
S7R	F	68.5	3.2	27.9	CI24RE (CS)	Exp 1 & 2
S8R	M	78.9	3.2	26.0	CI24RE (CS)	Exp 1
S9L	M	70.2	4.2	1.0	CI24RE (CS)	Exp 1 & 2
S10L	F	64.2	13.2	0.8	CI24R (CS)	Exp 2
S10R	F	64.2	1.5	12.4	CI24RE (CS)	Exp 1 & 2
S12L	M	81.2	2.3	3.0	CI24RE (CS)	Exp 1 & 2

All testing used a monopolar stimulation (MP 1+2) mode and biphasic pulse trains with a phase duration of 25 μs and an interphase interval of 8 μs. For psychophysical testing, a Nucleus® Freedom processor (Cochlear Corporation, Englewood, CO) was used. The experiments were controlled by MATLAB programs interfacing with the NIC II research software. For speech testing, a laboratory owned processor that was the same as what the subject wore everyday was used. Speech testing material was delivered by a loudspeaker 1 meter from the subject at 0 degree azimuth in a double-walled sound booth.

### Experiment 1

For each ear, two to three electrodes were tested for spatial selectivity and detection thresholds for biphasic pulse trains of three categories: (a) low rate and long duration, (b) high rate and short duration, and (c) high rate and long duration. The selection of the electrodes was arbitrary. The parameter details of the stimuli are given in Table 2. There were two stimuli under each category. Note that 40-pps and the 15.625-ms stimuli had the same number of pulses, so did the 80-pps and 31.25-ms stimuli. The 500-ms and 900-pps stimulus was chosen because it is commonly used clinically for measuring threshold level. Detection thresholds for the 6 stimuli were first estimated using the method of adjustment (MOA), where the subjects were instructed to increase the level of the stimulus sufficiently high so that the stimulus was confidently perceived and then bracket the level until the stimulus was just detectable. Current adjustments were made in 25, 5, or 1 CLU (clinical level unit). The MOA thresholds helped to set the starting point of the following adaptive procedures. The 3-interval forced choice paradigm (3IFC) started at 20 CLU above the MOA thresholds, and the level of the signal interval adapted following a 2-down 1-up criterion. The adapting step size was 10 CLU for the first reversal, 5 CLU for the second, 2 CLU for the third, and 1 CLU for the rest of the seven reversals. The levels at the last six reversals were averaged and the mean was taken as the threshold.

**Table 2:** Stimuli parameters.

Stimuli category	Stimulation rate (pps)	Duration (ms)	Number of pulses
Low rate	40	250	10
	80	250	20
Short and high rate	640	15.625	10
	640	31.25	20
Long and high rate	640	250	160
	900	500	450

A forward-masking paradigm was used to measure spatial selectivity of neural excitation. It is a commonly used method for measuring neural interactions between channels, where a masker signal is presented forward in time followed by a short gap and then the probe signal [16–23]. If the masker and probe excited an overlapping populations of neurons, the neurons that had been activated by the forward masker would be less responsive to the probe causing the probe threshold to elevate. The amount of probe threshold elevation, quantified as forward masking, was measured as a function of the spatial separation between the masker and probe. If probe threshold were elevated by a masker spatially separated from the probe (i.e., off-site masker), it would indicate neural interaction between the probe and masker sites. The masker and probe stimuli used a high rate of 900 pps to ensure that, if there is any interaction between the masker and probe site, the interaction will cause the probe threshold to elevate. The forward masker was 300 ms long, followed by a 10-ms gap, and a 20-ms probe. The location of the probe was fixed while seven maskers were positioned around the probe such that three were apical to the probe, one on the same location as the probe, and three were basal to the probe (if spatially allowed). The threshold level of the probe and masker signals was first estimated using MOA. To determine the maximum comfortable level of the probe and maskers, the subject was instructed to find the level that corresponded to a 9 on a loudness scale of 1 to 10. The level of the maskers was initially set at 50% of the respective DR. The on-site maskers (the maskers that were on the same site as the probes) were loudness balanced to each other. The off-site maskers were then loudness balanced to the corresponding on-site masker. Finally, the seven maskers for a given probe location were swept in both directions before fine adjustments were made. Because the masker levels were loudness balanced, it was possible that a masker site in poorer condition would require a higher current level to achieve the designated loudness, thus producing greater excitation at the probe site compared to a masker with a lower absolute current level. The possible unequal excitation produced by the loudness-balanced maskers would produce some noise in the masking patterns. A similar caveat exists for using a forward-masked psychophysical tuning curve, where it is not possible to equate excitation produced by the probes at different tonotopic locations or across ears [22].

Detection threshold of the probe was first measured without masker and then with a spatially-varying masker using 3IFC. One of the intervals chosen at random contained the masker-probe signal while the other two intervals contained the masker only. The level of the probe started at 50% DR and adapted using a 2-down 1-up criterion. The amount of threshold elevation in dB was quantified as forward masking, which was calculated for each masker-probe separation condition. Each data point on the forward-masking function was then divided by peak masking (forward masking at 0 masker-probe separation) to remove the across-probe differences in masking decay at 10 ms [22]. The rate at which the normalized forward masking decays with the increasing probe-masker separation (slope) reveals the extent to which neural excitation produced by the probe overlaps with that produced by the masker (s). The forward masking slopes were calculated for both the apical and basal sides of the function by fitting a linear line to the amount of forward masking against the exact mm masker-probe spacing for each masker-probe separation condition (electrode spacing is not uniform for CI24RE and CI512). If forward masking decayed with increasing masker-probe separation in mm, the slope would be negative. It should be noted that a linear fit to the arbitrarily chosen masker-probe separations may not best describe the shape of all functions.

### Experiment 2

Based on the results of Experiment 1, the strongest threshold-predictor for spatial selectivity was identified (i.e., 80-pps thresholds). For each tested, the threshold was then measured for the entire electrode array using MOA. The electrodes were rank ordered based on their thresholds. The electrode with the highest threshold was chosen without replacement from the array. If the electrode was immediately adjacent to any of the previously chosen ones, the electrode with the next highest threshold was considered until a total of five electrodes with high thresholds were identified. These electrodes were turned off in experimental map 1. Frequencies were automatically reallocated following deactivation. The deactivation gave rise to an experimental map that was otherwise identical to the subjects’ everyday-use clinical map in terms of levels, volume, sensitivity, stimulation rate, mode, speech processing strategy, and smart sound settings. When there were electrodes that had already been deactivated in the clinical map due to impedance issues or perception discomfort, those electrodes remained deactivated in the experimental map. In addition, a control condition (experimental map 2) was created, where 5 randomly chosen electrodes that were also not immediately adjacent to each other were deactivated. Note that some of the randomly chosen electrodes overlapped with those selected for experimental map 1. Six ears were tested for experimental map 2. The subjects were asked to switch back and forth between the clinical and the experimental maps that were loaded on the processor to determine whether a volume adjustment was needed for either one. No subject required a volume increase for the experimental maps or the clinical map.

The maps were evaluated in random order for speech recognition in quiet and in noise. The order was kept blind to the subject but not to the experimenter. A speech reception threshold (SRT) for CUNY sentences [24] was measured twice for each map. The sentences were meaningful utterances that differed in length. The noise was a white noise amplitude modulated at 4 Hz with a 100% modulation depth. The level of the noise was kept at 55 dB (A) SPL with a raised cosine ramp applied to the onset and offset. The noise was presented alone for 1.5 sec before the target sentence, during the target, and for 0.5 sec alone after the target. The subjects were instructed to repeat the target sentence to the experimenter. The level of the target sentence started at 20 dB above the noise and adapted in step size of 2 dB based on the subject’s response. Following a one-down one-up criterion, the SRT corresponded to the 50% correct point on the psychometric function. Because the sentence level was adapted, at low signal to noise ratios (SNRs), it is possible that the threshold was underestimated. Four lists of CUNY sentences were used for measuring one SRT. There had not been a case where the number of sentences in the four lists was not sufficient for obtaining 12 reversals. The SNRs at the last 6 reversals was averaged and taken as the SRT. Speech recognition in quiet was evaluated using the TIMITs sentences [25]. The TIMIT corpus contains recordings of 630 speakers of eight major dialects of phonetically rich American English. The sentences provide very limited contextual information. The root mean square values of all sentences were equalized and presented at a level of 65 dB (A) SPL. For each condition, two different sentence lists each containing 20 sentences were used. The number of words that the subject repeated correctly was used to calculate the percent correct score for a given condition.

## Results

### Experiment 1

**Fig 1:**
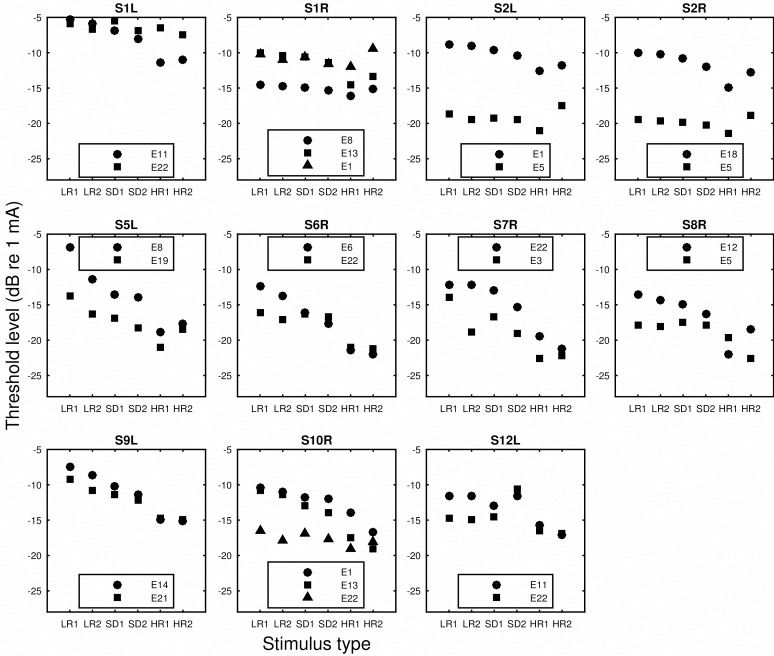
Thresholds for various stimulus types. Data collected from different ears are shown in separate panels. In each panel, thresholds are shown from left to right for the LR1 (40pps/250ms), LR2 (80pps/250ms), SD1 (640pps/15.625ms), SD2 (640pps/31.25ms), HR1 (640pps/250ms), and HR2 (900pps/500ms) stimulus type. Symbols indicate stimulation sites.

**Fig 2:**
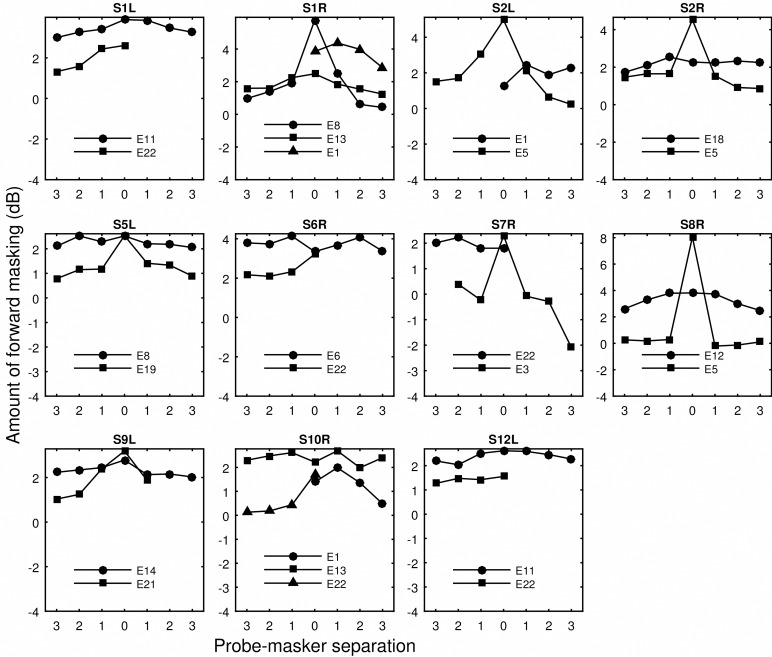
Raw forward masking as a function of probe-masker separation. Data collected from different ears are shown in separate panels. Symbols indicate stimulation sites. Probe-masker separations (in number of electrodes) left to 0 indicate masker locations basal to the probe. Missing data indicate that the probe site is close to either the basal or apical end of the electrode array. The y axis shows the amount of forward masking (threshold elevation) in dB.

**Fig 3:**
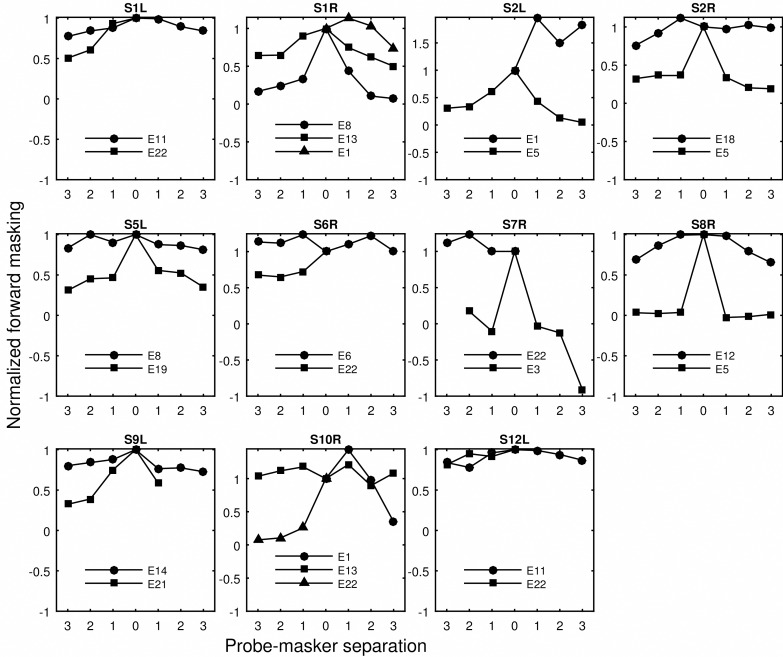
Normalized forward masking as a function of probe-masker separation. The same as Fig 2. The y axis shows normalized forward masking, i.e., the amount of forward masking at each probe-masker separation divided by forward masking produced by the on-site masker (0 mm separation).

The monopolar detection thresholds of the low rate and long duration (LR), high rate and short duration (SD), and high rate and long duration (HR) stimuli are shown in Fig 1. The thresholds were compared to the traditional measure of spatial selectivity of neural excitation, i.e., slope of the forward-masking functions. The raw forward masking functions are shown in Fig 2. In Fig 3, the amount of forward masking for each probe-masker separation condition was normalized to that at the 0 probe-masker separation. Shift of peak masking, that is, the maximum amount of masking occurring at probe-masker separation other than 0 mm, was often observed. The peak shifting was more often seen for masking functions that were shallow than those that were steep. The normalized forward masking did not always decay linearly with the probe-masker separation, although for simplicity of data analysis, spatial selectivity was quantified by fitting linear slopes to the function. The slopes fit to the basal side of the masking function did not significantly differ from those fit to the apical side (p > 0.05), as revealed by an unequal-sample-size *t* test not assuming equal variances. For probe sites that were masked for both the apical and basal directions, the basal and apical slopes were averaged and the average was used as the measure of spatial selectivity for neural excitation.

**Fig 4:**
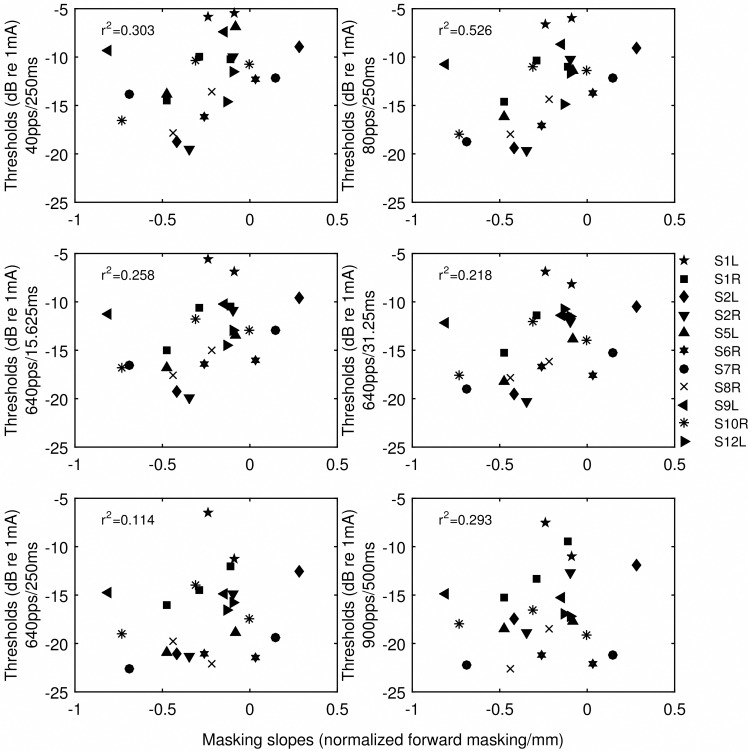
Scatter plots of thresholds versus the slopes of the normalized forward-masking functions. Threshold stimulus type is indicated by the y-axis label. Symbols indicate individual ears. The R^2^ values were those that were adjusted for examining two sources of variation in the general linear models. The R^2^ values indicate the strength of correlation between the threshold and masking slopes across sites and ears.

There were two sources of variance in the masking slopes, one between subjects and one between stimulation sites. Univariate general linear models were used to analyze whether the thresholds of various stimuli accounted for the between-subject and between-site variance of the masking slopes. The models used the forward-masking slopes as the dependent variable, subjects as the fixed variable, and threshold as the covariate. It is important to distinguish the between-subject and between-site effects, because only if thresholds explain the between-site variance in the masking slopes, would it provide rationale for using the thresholds to select stimulation sites in the processor maps (Experiment 2). The likelihood of each threshold explaining the between-site and between-subject variance in the masking slopes is indicated by the *p* values in Table 3. The total variance in the masking slopes accounted for by thresholds of various stimuli (R^2^) is also given in Table 3. Because both sources of variance were examined in the model, the correlation coefficient squared (R^2^) was adjusted for degree of freedom. The scatter plots of the thresholds of various stimuli against the masking slopes are shown in Fig 4 with the adjusted R^2^. The between-site variance in the masking slopes was accounted for by the thresholds of the two low-rate and the two short-duration stimuli. The general trend was that the predictive strength of threshold for spatial selectivity progressively decreased from the low-rate stimuli, to the short and high-rate stimuli, and to the high-rate and long stimuli. The strongest correlation was observed for thresholds detecting an 80-pps train.

**Table 3:** Univariate general linear models (dependent variable: forward masking slopes).

Stimuli category	Threshold predictor (pps/ms)	Between stimulation sites (*p* value)	Between subjects(*p* value)	Corrected model (*p* value)	R^2^	Adjusted R^2^
Low rate	40/250	*0*.*002*	0.233	0.141	0.636	0.303
	80/250	*< 0*.*0001*^a^	0.077	0.025	0.753	0.526
Short and high rate	640/15.625	*0*.*003*	0.281	0.18	0.613	0.258
	640/31.25	*0*.*005*	0.35	0.221	0.592	0.218
Long and high rate	640/250	0.05	0.681	0.651	0.419	0.114
	900/500	0.143	0.847	0.852	0.325	0.293

^a^ The underlined *p* values reached statistical significance after Bonferroni correction for multiple correlations (p < 0.008).

Pearson’s correlations were performed for all possible pairs of thresholds. The correlation coefficients are shown in Table 4. All correlations were significant after controlling for the family-wise Type I error by performing Bonferroni corrections (all *p* values < 0.003).

**Table 4:** Pearson's correlation between thresholds of various stimuli.

pps/ms	40/250	80/250	640/15.625	640/31.25	640/250	900/500
40/250	1	0.94	0.91	0.83	0.72	0.64
80/250		1	0.97	0.91	0.83	0.72
640/15.625			1	0.95	0.9	0.79
640/31.25				1	0.91	0.79
640/250					1	0.9
900/500						1

### Experiment 2

**Fig 5:**
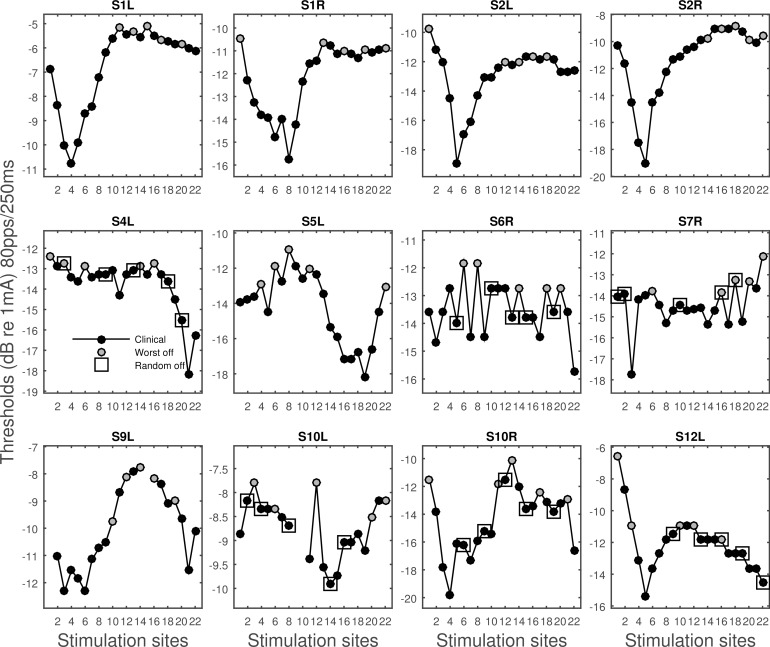
Thresholds of the 80-pps 250-ms pulse train as a function of stimulation sites. Stimulation sites deactivated in experimental map 1 are shown in grey. The five randomly chosen stimulation sites deactivated in experimental map 2 are indicated by the square symbol.

The 80-pps thresholds, which most strongly predicted the masking slopes, were measured for the whole electrode array for each ear, except for stimulation sites that had already been turned off in the subject’s clinical map (Fig 5). The across-site variation in the 80-pps thresholds was larger for some ears than the others, ranging from 0.6 to 2.8 dB in standard deviations with a mean of 1.67 dB. Compared to the thresholds measured in the clinical maps using the default 500-ms 900-pps parameters, the across-site variation in the 80-pps thresholds was significantly greater [t (11) = -2.9, p = 0.01].

**Fig 6:**
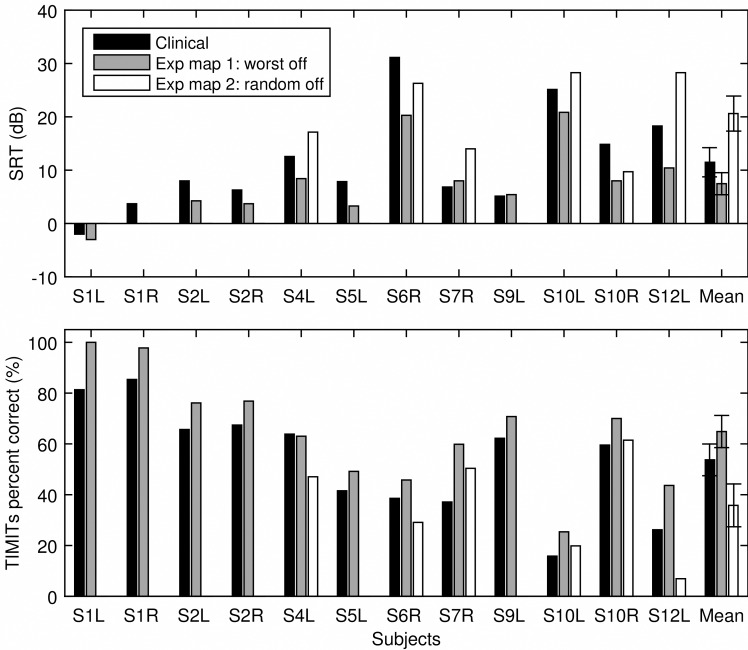
Speech recognition results. SRTs (top panel) and percent correct scores for TIMIT sentences in quiet (bottom panel) are shown for individual subjects as well as the group mean using the clinical map (black bars), experimental map 1 (grey bars), and experimental map 2 (white bars). The error bars represent standard error.

In Fig 5, the five stimulation sites with the highest 80-pps thresholds that were turned off in experimental map 1 were indicated by the grey color. Six subjects were tested for an experimental map 2 where five randomly chosen stimulation sites were turned off as indicated by the square symbol. Fig 6 shows speech recognition for TIMIT sentences in quiet and SRTs for CUNY sentence measured using the subjects’ clinical map and the two experimental maps. Repeated measure ANOVAs were performed to examine whether the experimental maps had a significant effect on SRTs and sentence recognition in quiet. Results showed that SRTs were significantly different across the map conditions [F (2) = 7.02, p = 0.01], so was recognition for the TIMIT sentences in quiet [F (2) = 6.22, p = 0.01]. Planned comparisons showed that relative to the clinical map, experimental map 1 significantly improved subjects’ SRTs [F (1) = 10.76, p = 0.02]. Although the group mean performance with experimental map 2 was worse than that with the clinical map, the difference was not statistically significant [F (1) = 0.943, p = 0.37]. Similarly for TIMIT sentences in quiet, performance with experimental map 1 significantly improved relative to that using the clinical map [F (1) = 9.18, p = 0.02]. The reduced performance with experimental map 2 relative to that with the clinical map was not statistically significant [F (1) = 0.733, p = 0.43]. On average, SRTs improved by 4.01 dB and recognition of the TIMIT sentences improved by 11.14 percentage points with the deactivation based on the 80-pps thresholds.

## Discussion

One of the greatest obstacles that limit the performance with cochlear implants is the lack of channel independence. This study examined the potential of using monopolar thresholds to estimate spatial selectivity of neural excitation in CI users. The results showed that the predictive power of the thresholds for spatial selectivity of neural excitation is dependent on parameters of the stimulus used for measuring the threshold. When neural responses to the stimulus is not likely limited by the temporal inhibitive factors such as refractoriness and adaptation, monopolar detection thresholds are strongly correlated with the slopes of the forward masking patterns. With the deactivation of the high-threshold stimulation sites estimated to produce channel interaction, speech recognition significantly improved both in quiet and in noise.

### Predictive power of monopolar thresholds

In the current study, three types of pulse trains contrasted in stimulation rate and duration (Table 2) were used to measure detection thresholds in monopolar stimulation. Thresholds for various stimulus types were then correlated with the slopes of the forward-masking functions to examine if they accounted for the between-subject and between-site variance in the masking slopes. Consistent with the hypothesis, the strongest predictor for spatial selectivity was threshold for the low-rate stimuli, i.e., 40 pps and 80 pps trains with a duration of 250 ms. From Figs 1 and 3, it can be seen that, within subjects, the stimulation site(s) that demonstrated a relatively broader masking pattern was always measured with a higher low-rate threshold. When the masking patterns were similar, their low-rate thresholds were also similar (S1L). However, when the threshold stimulus was increased in rate or duration, the differences in measured thresholds diminished (e.g., S5L, S6R, S7R, S9L), making these thresholds not as capable of differentiating sites that contrasted in spread of excitation. In other cases, the sign of the threshold difference between sites even reversed such as seen for S1L and S8R depending on stimulus type. For example, site 5 of S8R demonstrated a narrow masking pattern, which was reflected in its low low-rate threshold. However, one of the high-rate thresholds (HR1) measured at site 5 was found higher compared to site 12, the broad excitation site. Thresholds for the high-rate stimuli in these cases predicted a completely opposite spatial neural excitation patterns. The different across-site variation patterns in threshold produced stronger inter-correlation between some threshold pairs than others (Table 4).

The following provides some plausible explanations for the systematic change in the correlation strength between detection thresholds of the various stimulus types and spatial selectivity of neural excitation. At low rates, the interpulse intervals of these stimuli were likely long enough that neural responses to the stimuli did not involve any interpulse interaction. That is, the neurons would always be able to recover in time from their prior activity to respond to pulse that follows, based on what is known about the relative refractory period of the electrically stimulated auditory nerve [5, 6, 26]. The inhibitive effects of the sub-threshold pulses are also likely to be small given the large interpulse interval [7, 27]. Within a time period following stimulus onset, the number of spikes that single neurons generate following the low rates of stimulation would not be large enough for the neurons to adapt to their spiking rates [11, 12, 28]. Thresholds of the low-rate stimuli therefore were relatively free of any neural temporal response variables and were mostly subject to the spatial effects such as electrode-neuron distance, nerve loss or impedance. It is unclear however why the correlation with the masking patterns would be stronger for the 80-pps than the 40-pps thresholds.

When the pulses in the low-rate stimuli were pressed in time, such that the same number of pulses was presented in shorter time windows, i.e., 15.625 ms and 31.25 ms at 640 pps, neural responses to the stimulus become subject to interpulse interactions. If the high-rate stimulation continues for a long period of time, neural responses to the stimulus become subject to an additional factor of spike-rate adaptation or even central inhibition. With short interpulse intervals, excitability of the neurons can be reduced after they have fired to a preceding pulse, or have been desensitized by a sub-threshold one. Due to the differences in the absolute and relative refractory period of the auditory fibers, neural responses to high-rate pulse trains (400-2000 pps) typically exhibit an oscillating pattern in amplitudes [28–29]. With long stimulus duration, ECAP (electrically evoked compound action potential) amplitude can significantly reduce 30 ms into the stimulus at a rate of 500 pps, equivalent to stimulation of 15 pulses [28–29]. The more temporal factors that are involved in detection, the more “noisy” the threshold would be in reflecting any differences in the spatial spread of excitation for different electrodes. Further, as mentioned above, high-rate thresholds were often quite similar for stimulation sites that contrast greatly in spatial excitation patterns (Fig 1). In extreme cases, threshold was found lower at a broad stimulation site compared to that with narrow stimulation. This is perhaps because a broad excitation pattern (not in case of sparse neural survival), can release some of the temporal constrains on single fibers by providing a larger number of excitable neurons in response to high-rate stimulation. Previous findings [13] (and those observed in the current study) have suggested that detection threshold decreases more steeply with increasing rate with a broader stimulation pattern. ECAP growth functions were also found to be the steepest in the middle of the electrode array [30], where stimulation was often distant and broad. Broad stimulation may similarly reduce neural fatigue by exciting a large number of neurons thus lowering the amount of stimulation each neuron receives which in turn lowers their spiking rate [31]. Since a broad stimulation pattern can facilitate detection and lower threshold when stimulation is of high rate or long duration, these thresholds would not be as accurate for estimating the spatial excitation patterns. Threshold thus can be a complex function of the spatial factors, interpulse interactions (refractoriness and sub-threshold adaptation), and spike-rate adaptation, depending on stimulus parameters.

Taking together, the systematically weakened relationship between monopolar thresholds and the measure of spatial selectivity of neural excitation seems to be attributed to (1) the extent to which detection depends on the neural temporal response characteristics, and (2) the extent to which the spread of excitation facilitates detection when neural responses are inhibited by refractoriness and adaptation.

### Monopolar versus focused stimulation

The weak or lack of correlation between monopolar thresholds and speech recognition and other psychophysical measures [32–33] has been attributed to the possibility that monopolar thresholds are not sensitive to the conditions local to the stimulation sites because the current is returned to remote ground electrodes. Previous studies have demonstrated that the relationship between thresholds and psychophysical tuning curves can be improved if a more focused stimulation mode is used where a portion of the current is returned to the intracochlear electrodes adjacent to the active ones. Bierer and colleagues [33–34] showed that psychophysical or EABR (electrically evoked auditory brainstem response) thresholds measured with partial tripolar configuration more strongly predicted the slopes of the apical side of forward-masked psychophysical tuning curves than those measured with monopolar stimulation. The results of the current study suggest that the weak correlation between the monopolar thresholds and the spread of excitation measure might also be due to stimulus parameters. It remains to be tested whether the predictive power of the low-rate thresholds for spread of excitation measures improves when a more focused stimulation mode is used.

### Across-site variation pattern in the low-rate thresholds

The 80-pps threshold, which was the strongest predictor for spread of neural excitation, was measured across the stimulation sites for all ears. On average, the across-site variation in the 80-pps thresholds was significantly greater compared to that in the clinical thresholds that used the default 900-pps 500-ms parameters. This could be partly due to the diminished differences in thresholds when threshold stimulus is increased in rate or duration. As discussed above, a broad stimulation pattern facilitates detection by providing more excitable neurons that are not in an inhibited status, thus reducing the threshold difference compared to sites that receive narrow stimulation. It should be noted however that interpolation of threshold levels across electrodes that is often performed in clinical fitting can also reduce the across-site variation in the clinical thresholds. It would also be interesting to compare across-site variation patterns in the monopolar low-rate thresholds and focused thresholds.

### The effect of deactivation of stimulation sites

Experiment 2 examined the effect of deactivating stimulation sites based on the 80-pps thresholds (experimental map 1) and stimulation sites that were randomly chosen (experimental map 2). Deactivation of the stimulation sites resulted in an automatic reallocation of frequencies to the remaining active electrodes, such that the bandwidths assigned to the remaining stimulation sites were broadened. Using the experimental map that deactivated the five electrodes measured with the highest 80-pps thresholds, the majority of the subjects achieved improved SRTs and speech recognition in quiet. The averaged group performance was significantly better using experimental map 1 than the clinical map that had all electrodes active. Note that the distribution of the deactivated electrodes did not seem to contribute to performance. Deactivating more electrodes in the apical region did not produce performance improvement any different than deactivating more electrodes in the basal region, which suggests that it was not the tonotopic location but the condition at the location that contributed to speech recognition in ears with pathology. Random deactivation was detrimental in many cases, but for some subjects, performance did improve although the improvement was smaller than that resulted from turning off the five electordes with the highest thresholds. For those subjects who benefited from the random deactivation, it seems that simply increasing the distance between any pair of active electrodes improved performance, which could suggest overall excessive channel interaction. The group mean with random deactivation was worse than that using the clinical map, but the difference was not significant perhaps due to the small sample size tested for experimental map 2. The results suggested that caution should be used if random deactivation was to be used, as it might be detrimental for some subjects, or it might not be as effective as targeting sites that produce the greatest interaction.

If channel interaction is present, a given neural population would receive and process a wider than expected band of speech information, because the neurons must process pulse trains delivered by the closest electrode as well as those imposed by the distant ones. This however, is not equivalent to simply stimulating the same subpopulation of neurons with a broadened frequency band, because with deactivation, the fewer stimulation sites each assigned with a relatively wide band produces better performance than using all stimulation sites that interact. These results seem to suggest that channel interaction might cause distortions in neural coding of the speech signal. When a subpopulation of neurons receives modulated pulse trains from multiple electrodes, the effective rate at which the neurons are being stimulated could be far greater than the single-electrode rate of 900 pps. As discussed earlier, multiple inhibitive factors can cause the neurons to reduce excitability in response to high-rate stimulation. If the pulses from remote electrodes are sub-threshold to the subpopulation of neurons, sub-threshold adaptation could occur preventing the neurons from responding to the pulses delivered by the closest electrode. The possible spatial summation of stimulation could explain why a full-array activation might not necessarily be optimal. The number of electrodes needed to be deactivated for optimal speech results is perceivably different for each ear given the individual differences in pathology and electrode position patterns. The fact that some subjects benefited from random deactivation suggests that for those individuals turning off five electrodes might not be sufficient. Because the thresholds were only predictive of the between-electrode variations in spread of excitation and not the between-subject variation, it has not been possible to determine the amount of deactivation needed based on the absolute threshold levels. The effects of deactivation were also acute in that the subjects were tested for the experimental maps without adapting to the changes in frequency allocation. It is possible that a greater effect would be observed if some experience were gained with the experimental maps.

There has not been consistent results indicating a relationship between measures of spatial selectivity of neural excitation and speech recognition across subjects [30]. The slopes of the spatial tuning curves averaged from three tonotopic locations for example did not correlate with the subjects’ speech recognition performance [16]. One explanation for the lack of correlation is that the sampled locations were not always representative of the spatial neural excitation pattern on the entire array. Another possibility is that the across-subjects correlation was masked by subject variables such as cognitive abilities.

Site-selection strategies based on other psychophysical measures such as amplitude modulation detection thresholds [35–36] and CT scans [37–38] have also shown to improve speech recognition. It is yet to be examined whether these measures share the same underlying mechanisms as detection thresholds. For example, it is unknown whether the difficulty with detecting amplitude modulation is related to the detrimental effect of current spread or other variables that might also be important for speech recognition with cochlear implants. Focused stimulation has also been proposed to decrease channel interaction, although it has not consistently provided benefit relative to monopolar stimulation [39–40]. Different from electrode deactivation, the outcome of focused stimulation may depend on whether the small-size subgroup of neurons is healthy and whether they are able to process normal stimulation rates without excessive adaptation.

## References

[1] Friesen LM, Shannon RV, Baskent D, Wang X. Speech recognition in noise as a function of the number of spectral channels: Comparison of acoustic hearing and cochlear implants. J Acoust Soc Am. 2001; 110: 1150–1163. 11519582 10.1121/1.1381538

[2] Fu QJ, Nogaki G. Noise susceptibility of cochlear implant users: The role of spectral resolution and smearing. J Assoc Res Otolaryngol. 2005; 6: 19–27. 10.1007/s10162-004-5024-3 15735937 PMC2504636

[3] Nadol JB Jr. Patterns of neural degeneration in the human cochlea and auditory nerve: Implications for cochlear implantation. Otolaryngol Head Neck Surg. 1997; 117: 220–228. 9334769 10.1016/s0194-5998(97)70178-5

[4] Long CJ, Holden TA, McClelland GH, Parkinson WS, Shelton C, Kelsall DC, et al Examining the electro-neural interface of cochlear implant users using psychophysics, CT scans, and speech understanding. J Assoc Res Otolaryngol. 2014; 15: 293–304. 10.1007/s10162-013-0437-5 24477546 PMC3946134

[5] Miller CA, Abbas PJ, Robinson BK. Response properties of the refractory auditory nerve fiber. J Assoc Res Otolaryngol. 2001; 2: 216–232. 10.1007/s101620010083 11669395 PMC3201673

[6] Cartee LA, van den Honert C, Finley CC, Miller RL. Evaluation of a model of the cochlear neural membrane. I. Physiological measurement of membrane characteristics in response to intrameatal electrical stimulation. Hear Res. 2000; 146: 143–152. 10913891 10.1016/s0378-5955(00)00109-x

[7] Miller CA, Woo J, Abbas PJ, Hu N, Robinson BK. Neural masking by sub-threshold electric stimuli: Animal and computer model results. J Assoc Res Otolaryngol. 2011; 12: 219–232. 10.1007/s10162-010-0249-9 21080206 PMC3046329

[8] Cohen LT. Practical model description of peripheral neural excitation in cochlear implant recipients: 5. Refractory recovery and facilitation. Hear Res. 2009; 248: 1–14. 10.1016/j.heares.2008.11.007 19110048

[9] Cartee LA, Miller CA, van den Honert C. Spiral ganglion cell site of excitation I: Comparison of scala tympani and intrameatal electrode responses. Hear Res. 2006; 215:10–21. 10.1016/j.heares.2006.02.012 16624511

[10] Middlebrooks JC. Effects of cochlear-implant pulse rate and inter-channel timing on channel interactions and thresholds. J Acoust Soc Am. 2004; 116: 452–468. 15296005 10.1121/1.1760795

[11] Killian MJP, Klis SFL, Smoorenburg GF. Adaptation in the compound action potential response of the guinea pig VIIIth nerve to electrical stimulation. Hear Res. 1994; 81: 66–82. 7737931 10.1016/0378-5955(94)90154-6

[12] Zhang F, Miller CA, Robinson BK, Abbas PJ, Hu N. Changes across time in spike rate and spike amplitude of auditory nerve fibers stimulated by electric pulse trains. J Assoc Res Otolaryngol. 2007; 8: 356–372. 10.1007/s10162-007-0086-7 17562109 PMC2538432

[13] Zhou R, Abbas PJ, Assouline JG. Electrically evoked auditory brainstem response in peripherally myelin-deficient mice. Hear Res. 1995; 88: 98–106. 8576009 10.1016/0378-5955(95)00105-d

[14] Cohen LT. Practical model description of peripheral neural excitation in cochlear implant recipients: 5. Refractory recovery and facilitation. Hear Res. 2009; 248: 1–14. 10.1016/j.heares.2008.11.007 19110048

[15] Zhou N, Pfingst BE. Evaluating multipulse integration as a neural-health correlate in human cochlear-implant users: Relationship to spatial selectivity. J Acoust Soc Am. 2016; 140: 1537–1547. 27914377 10.1121/1.4962230 PMC5392072

[16] Nelson DA, Kreft HA, Anderson ES, Donaldson GS. Spatial tuning curves from apical, middle, and basal electrodes in cochlear implant users. J Acoust Soc Am. 2011; 129: 3916–3933. 10.1121/1.3583503 21682414 PMC3135148

[17] Chatterjee M, Shannon RV. Forward masked excitation patterns in multielectrode electrical stimulation. J Acoust Soc Am. 1998; 103: 2565–2572. 9604350 10.1121/1.422777

[18] Boex C, Kos MI, Pelizzone M. Forward masking in different cochlear implant systems. J Acoust Soc Am. 2003; 114: 2058–2065. 14587605 10.1121/1.1610452

[19] Kwon BJ, van den Honert C. Effect of electrode configuration on psychophysical forward masking in cochlear implant listeners. J Acoust Soc Am. 2006; 119: 2994–3002. 16708955 10.1121/1.2184128

[20] Dingemanse JG, Frijns JH, Briaire JJ. Psychophysical assessment of spatial spread of excitation in electrical hearing with single and dual electrode contact maskers. Ear Hear. 2006; 27: 645–657. 10.1097/01.aud.0000246683.29611.1b 17086076

[21] Hughes ML, Stille LJ. Psychophysical versus physiological spatial forward masking and the relation to speech perception in cochlear implants. Ear Hear. 2008; 29: 435–452. 10.1097/AUD.0b013e31816a0d3d 18344869 PMC2467511

[22] McKay CM. Forward masking as a method of measuring place specificity of neural excitation in cochlear implants: A review of methods and interpretation. J Acoust Soc Am. 2012; 131: 2209–2224. 10.1121/1.3683248 22423717

[23] McKay CM, Chandan K, Akhoun I, Sicilliano C, Kluk K. Can ECAP measures be used for totally objective programming of cochlear implants?. J Assoc Res Otolaryngol. 2013; 14: 879–890. 10.1007/s10162-013-0417-9 24048907 PMC3825020

[24] Boothroyd A, Hanin L, Hnath T. A sentence test of speech perception: Reliability, set equivalence, and short term learning New York City: City University of New York; 1986.

[25] Garofolo J, Lamel L, Fisher W, Fiscus J, Pallett D, Dahlgren N, et al TIMIT acoustic–phonetic continuous speech corpus LDC93S1 Philadelphia: Linguistic Data Consortium; 1993.

[26] Stypulkowski PH, van den Honert C. Physiological properties of the electrically stimulated auditory nerve. I. Compound action potential recordings. Hear Res. 1984; 14: 205–223. 6480510 10.1016/0378-5955(84)90051-0

[27] Boulet J, White M, Bruce IC. Temporal considerations for stimulating spiral ganglion neurons with cochlear implants. J Assoc Res Otolaryngol. 2015; 17: 1–17. 10.1007/s10162-015-0545-5 PMC4722016 26501873

[28] Wilson BS, Finley CC, Lawson DT, Zerbi M. Temporal representations with cochlear implants. Am J Otol. 1997; 18: S30–S34. 9391587

[29] Hughes ML, Castioni EE, Goehring JL, Baudhuin JL. Temporal response properties of the auditory nerve: Data from human cochlear-implant recipients. Hear Res. 2012; 285: 46–57. 10.1016/j.heares.2012.01.010 22326590 PMC3299843

[30] Tang Q, Benitez R, Zeng FG. Spatial channel interactions in cochlear implants. J Neural Eng. 2011; 8: 046029 10.1088/1741-2560/8/4/046029 21750370 PMC3190971

[31] Woo J, Miller CA, Abbas PJ. The dependence of auditory nerve rate adaptation on electric stimulus parameters, electrode position, and fiber diameter: A computer model study. J Assoc Res Otolaryngol. 2010; 11: 283–296. 10.1007/s10162-009-0199-2 20033248 PMC2862915

[32] Pfingst BE, Xu L, Thompson CS. Across-site threshold variation in cochlear implants: Relation to speech recognition. Audiol Neurootol. 2004; 9: 341–352. 10.1159/000081283 15467287 PMC1450110

[33] Bierer JA, Faulkner KF. Identifying cochlear implant channels with poor electrode-neuron interface: Partial tripolar, single-channel thresholds and psychophysical tuning curves. Ear Hear. 2010; 31: 247–258. 10.1097/AUD.0b013e3181c7daf4 20090533 PMC2836401

[34] Bierer JA, Faulker KF, Tremblay KL. Identifying cochlear implant channels with poor electrode-neuron interface: Electrically-evoked auditory brainstem responses measured with the partial tripolar configuration. Ear Hear. 2011; 32: 436–444. 10.1097/AUD.0b013e3181ff33ab 21178633 PMC3082606

[35] Zhou N, Pfingst BE. Psychophysically-based site selection coupled with dichotic stimulation improves speech recognition in noise with bilateral cochlear implants. J Acoust Soc Am. 2012; 132: 994–1008. 10.1121/1.4730907 22894220 PMC3427365

[36] Garadat SN, Zwolan TA, Pfingst BE. Using temporal modulation sensitivity to select stimulation sites for processor maps in cochlear implant listeners. Audiol Neurootol. 2013; 18: 247–260. 10.1159/000351302 23881208 PMC3874548

[37] Labadie RF, Noble JH, Hedley-Williams AJ, Sunderhaus LW, Dawant BM, Gifford RH. Results of postoperative, CT-based, electrode deactivation on hearing in prelingually deafened adult cochlear implant recipients. Otol Neurotol. 2016; 37: 137–45 10.1097/MAO.0000000000000926 26719955 PMC4712086

[38] Noble JH, Hedley-Williams AJ, Sunderhaus L, Dawant BM, Labadie RF, Camarata SM, et al Initial results with image-guided cochlear implant programming in children. Otol Neurotol. 2016; 37: E63–E69. 10.1097/MAO.0000000000000909 26756157 PMC4849538

[39] Pfingst BE, Franck KH, Xu L, Bauer EM, Zwolan TA. Effects of electrode configuration and place of stimulation on speech perception with cochlear prostheses. J Assoc Res Otolaryngol. 2001; 2: 87–103 10.1007/s101620010065 11550528 PMC3201186

[40] Srinivasan AG, Padilla M, Shannon RV, Landsberger DM. Improving speech perception in noise with current focusing in cochlear implant users. Hear Res. 2013; 299: 29–36. 10.1016/j.heares.2013.02.004 23467170 PMC3639477

